# Associated Factors with Biochemical Hypoglycemia during an Oral Glucose Tolerance Test in a Chinese Population

**DOI:** 10.1155/2017/3212814

**Published:** 2017-08-22

**Authors:** Xiaoling Cai, Xueyao Han, Xianghai Zhou, Lingli Zhou, Simin Zhang, Linong Ji

**Affiliations:** Endocrine and Metabolism Department, Peking University People's Hospital, Beijing 100044, China

## Abstract

**Aim:**

To find the association between biochemical hypoglycemia on a 2-hour screening oral glucose tolerance test (OGTT) and insulin resistance.

**Method:**

Subjects of this study were sampled from the China National Diabetes and Metabolic Disorders Study that was conducted during 2007 and 2008. Blood samples were drawn at 0, 30, and 120 minutes after the glucose load. Biochemical hypoglycemia was defined as 2-hour glucose < 3.0 mmol/l.

**Results:**

In total, 26,606 participants were included, and 141 participants were diagnosed with biochemical hypoglycemia on a 2-hour OGTT. Compared to participants with normal glucose tolerance (NGT), participants with biochemical hypoglycemia presented with a younger age, lower BMI, lower levels of fasting glucose, and lower levels of 30-minute and 2-hour OGTT glucose. In terms of insulin resistance, participants with biochemical hypoglycemia showed higher levels of Matsuda ISI. In terms of *β*-cell function, participants with biochemical hypoglycemia showed higher levels of Stumvoll early and late indexes. A multivariate regression analysis indicated that higher levels of Matsuda ISI and higher levels of Stumvoll early and late indexes were associated with biochemical hypoglycemia independently.

**Conclusion:**

This study indicated that biochemical hypoglycemia might be associated with lower levels of insulin resistance but higher levels of *β*-cell function in a Chinese population.

## 1. Introduction

In a population-based screening for type 2 diabetes, although biochemical hypoglycemia is not a common finding on oral glucose tolerance tests (OGTTs), it really exists in some subjects. Previously, it was thought to be potentially linked with an increased type 2 diabetes risk [1], and the possible mechanism of biochemical hypoglycemia was considered the link to insulin resistance. As indicated by several studies, insulin resistance leads to basal-fasting hyperinsulinemia, which may result in the insensitivity of beta-cell secretion. This insensitivity leads to a lower first-phase insulin secretion to the rising postprandial glucose but a subsequent exaggerated compensatory second-phase insulin secretion, which might result in hypoglycemia [2, 3].

So far, there are very little data considering the association between biochemical hypoglycemia during an OGTT and insulin resistance [4, 5]. Results from these studies indicated that postchallenge hypoglycemia might be an indicator of future diabetes when it occurs in patients without overt diabetes or impaired glucose tolerance (IGT). However, Parekh et al. recently reported on an analysis from a United Kingdom population. This analysis indicated that biochemical hypoglycemia during an OGTT in the absence of diabetes or impaired glucose regulation (IGR) was not associated with insulin resistance [6]. They also indicated that biochemical hypoglycemia on an OGTT may not potentially be an early indicator of future diabetes. Another recently published study suggested that biochemical hypoglycemia might be associated with insulin secretion rather than insulin resistance [7].

Is biochemical hypoglycemia on an OGTT associated with insulin resistance in a Chinese population? The aim of this study was to find the association between biochemical hypoglycemia on a 2-hour screening OGTT and insulin resistance in a Chinese population.

## 2. Methods

### 2.1. Subjects

Subjects of this study were sampled from the China National Diabetes and Metabolic Disorders Study that was conducted during 2007 and 2008 [8]. The rationale and design of the China National Diabetes and Metabolic Disorders Study have been described previously [8]. Inclusion criteria for this study were as follows: (1) participants with available OGTT data and (2) participants with NGT. Participants who had known diabetes or who met the diagnostic criteria for diabetes or IGR were excluded from this analysis. The Institutional Review Board and Ethics Committee at each participating institution approved the study protocol. Each participant provided written informed consent.

### 2.2. Data Collection

The anthropometrics and biochemical measurements were described previously as follows [8]. Participants were instructed to maintain their usual physical activities and diets for at least 3 days before the OGTT. After at least 10 hours of overnight fasting, venous blood specimens were collected in vacuum tubes containing sodium fluoride to measure circulating biochemical variables. Participants with no history of diabetes were given a standard 75 g glucose solution. Blood samples were drawn at 0, 30, and 120 minutes after the glucose load to measure circulating concentrations of glucose and insulin. Plasma glucose was measured by applying a hexokinase enzymatic method, and serum immune reactive insulin was analyzed by applying radioimmunoassay (North Biotechnology Institute, Beijing, China; intra- and interassay coefficient of variation <5%). Fasting serum levels of triglyceride and high-density lipoprotein cholesterol were measured as recently reported [8].

### 2.3. Assessment of Biochemical Hypoglycemia

Biochemical hypoglycemia was defined as OGTT 2-hour glucose (Glu120) < 3.0 mmol/l. The cut point of 3.0 mmol/l for blood glucose was recommended by the definition of hypoglycemia according to American Diabetes Association (ADA) guidelines.

### 2.4. Calculation of Estimates of Insulin Release and Sensitivity Index

1. The Matsuda's insulin sensitivity index (Matsuda ISI) was calculated as 10,000/(FPG × 18 × Ins0 × mean glucose level in OGTT × 18 × mean insulin level in OGTT)^1/2^ [9].
2. Stumvoll's early *β*-cell function (EPIR) was calculated as 2032 + (4.681 × Ins0 × 6) − (135.0 × Glu120) + (0.995 × Ins120 × 6) + (27.99 × BMI) − (269.1 × FPG)  [10].
3. Stumvoll's late *β*-cell function (LPIR) was calculated as 277 + (0.8000 × Ins0 × 6) − (42.79 × Glu120) + (0.321 × Ins120 × 6) + (5.338 × BMI)  [10].

### 2.5. Statistical Analysis

All statistical analyses were performed using SPSS software, version 19.0. Data were expressed as mean ± SD (standard deviation) or as median with upper and lower quartiles. Continuous variables were compared by using a one-way analysis of variance (ANOVA) test, while a frequency of dichotomous variables was performed by *χ*^2^ analysis. Multivariable logistic regression analyses were applied to assess the relationships between biochemical hypoglycemia and Matsuda ISI, early *β*-cell function, and late *β*-cell function. A two-sided *p* ≤ 0.05 was considered significant.

## 3. Results

### 3.1. Prevalence of Biochemical Hypoglycemia

In total, 26,606 participants were included in this study analysis. Of these participants, 141 of them were diagnosed with biochemical hypoglycemia on a 2-hour OGTT with a prevalence 0.53%, and 26,465 of them were diagnosed with NGT. Patients with IGR, as well as diabetes, were excluded from this analysis.

### 3.2. Clinical Characteristics of Patients with Biochemical Hypoglycemia

The average age of the patients with biochemical hypoglycemia was 42.5 ± 13.1 years, and the average BMI of the patients was 22.8 ± 3.2 kg/m^2^. The average FPG, Glu30 level, and Glu120 level was 4.62 ± 0.65 mmol/l, 7.82 ± 2.07 mmol/l, and 2.92 ± 0.07 mmol/l, respectively. The average fasting insulin secretion, Ins30 level, and Ins120 level was 7.4 ± 4.6 *μ*U/ml, 53.0 ± 37.6 *μ*U/ml, and 16.3 ± 19.1 *μ*U/ml, respectively.

### 3.3. Comparisons of Clinical Characteristics between Participants with Biochemical Hypoglycemia and Those with NGT

Compared to participants with NGT, participants with biochemical hypoglycemia showed older age, lower BMI, and lower levels of fasting glucose, Glu30, and Glu120. In terms of insulin secretion, participants with biochemical hypoglycemia showed higher secretion levels of Ins30 but lower secretion levels of Ins120. Details are shown in Tables 1 and 2.

### 3.4. Comparisons of Insulin Resistance and Beta-Cell Function

**Figure 1:**
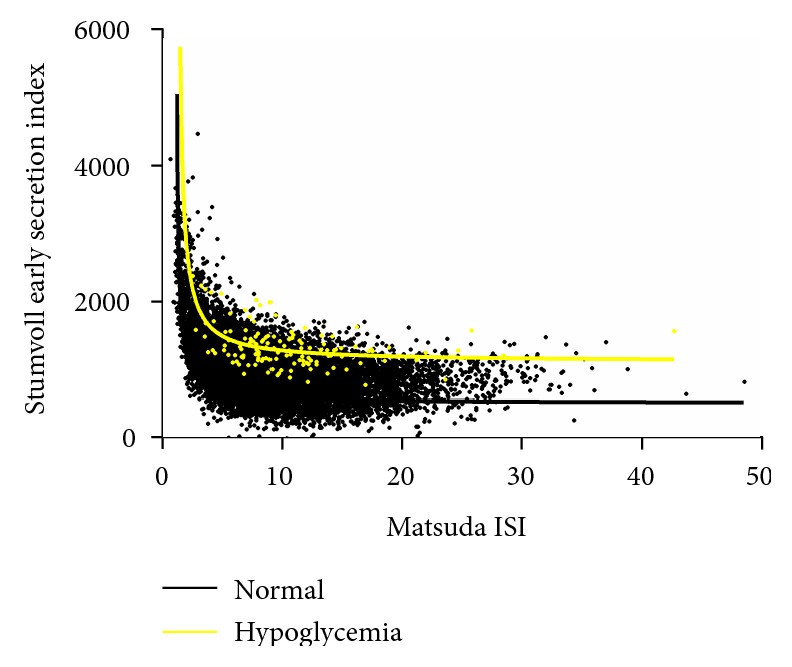
The insulin secretion of early stage and insulin sensitivity levels between participants with biochemical postprandial hypoglycemia and NGT.

**Figure 2:**
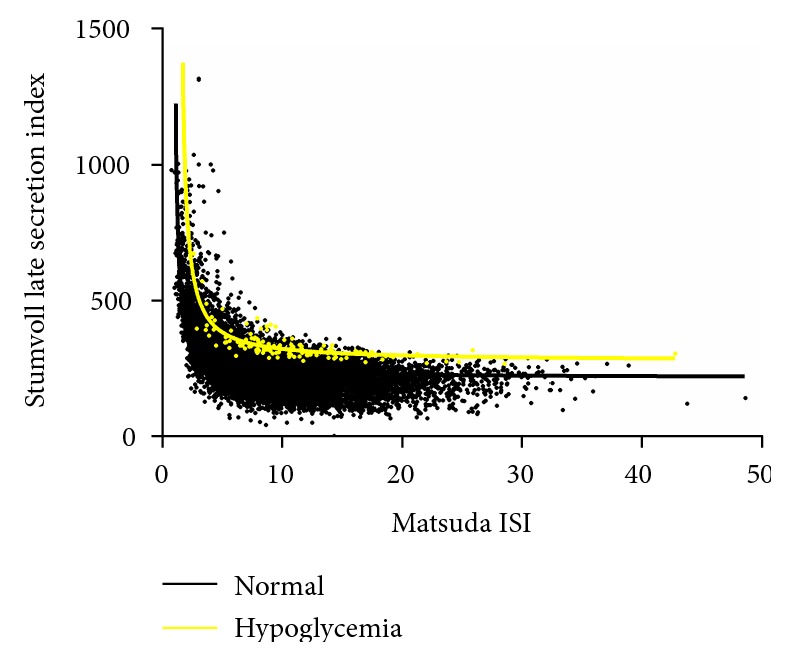
The insulin secretion of late stage and insulin sensitivity levels between participants with biochemical postprandial hypoglycemia and NGT.

Compared to participants with NGT, participants with biochemical hypoglycemia showed higher levels of Matsuda ISI, which may indicate lower levels of insulin resistance. In terms of *β*-cell function, participants with biochemical hypoglycemia showed higher levels of Stumvoll early and late indexes, which may indicate higher levels of *β*-cell function, both in the early and late stages. Details are shown in Table 2. A multivariate logistic regression analysis also indicated that higher levels of Matsuda ISI and higher levels of Stumvoll early and late indexes, were associated with biochemical hypoglycemia. Details are shown in Table 3. Figures 1 and 2 show the early and late stages of insulin secretion, as well as the insulin sensitivity levels between participants with biochemical postprandial hypoglycemia and NGT. These levels indicated that, with the same level of insulin sensitivity, participants with postprandial hypoglycemia had more insulin secretion both in the early and late stages compared to participants with NGT.

## 4. Discussion

Biochemical hypoglycemia is a relatively rare finding on OGTTs; therefore, little attention has been paid to postchallenge biochemical hypoglycemia compared to hyperglycemia. According to this cross-sectional analysis based on such a large Chinese population, the prevalence of postchallenge biochemical hypoglycemia was 0.53%. Results from this study indicated that, compared to participants with NGT, participants with biochemical hypoglycemia had higher levels of insulin sensitivity, as well as higher levels of *β*-cell function, both in the early and late stages.

The insulin sensitivity was evaluated as the level of Matsuda ISI, a standard figure for evaluating insulin resistance. Studies focusing on the insulin resistance and dysglycemia suggested that, compared to participants with NGT, patients with IGT or impaired fasting glycemia (IFG) had higher levels of HOMA-IR, which indicated that patients with postchallenge hyperglycemia tended to be more insulin resistant. On the other hand, the previous studies on postchallenge hypoglycemia also suggested that, compared to participants with NGT, patients with postchallenge hypoglycemia had higher levels of HOMA-IR and might be associated subsequently with IGT and diabetes. Arii et al. [4] reported on a case of a patient with reactive hypoglycemia. This patient was insulin resistant as demonstrated by the use of a euglycemic hyperinsulinemic glucose clamp; therefore, after treatment with pioglitazone to decrease insulin resistance, the patient showed a normal glucose level. Tamura et al. [5] reported on another case regarding an older patient with postprandial reactive hypoglycemia. This patient was diagnosed as having IGT. After treatment with acarbose, the patient no longer suffered from hypoglycemia. According to previous data, the occurrence of postchallenge hypoglycemia might be an indicator of early beta-cell dysfunction and insulin resistance.

On the contrary, Tamburrano et al. [11] performed a euglycemic hyperinsulinemic glucose clamp on 20 patients with symptoms suggesting reactive hypoglycemia, and the results suggested that increased insulin sensitivity represents a feature of idiopathic reactive hypoglycemia. Parekh et al. [6] recently indicated that biochemical hypoglycemia during an OGTT in the absence of diabetes or IGR was not associated with insulin resistance based on a United Kingdom multiethnic population. They also indicated that biochemical hypoglycemia might be associated with more favorable glycemic risk profiles than IGR and NGT. In addition, results from our study indicated that, compared to participants with NGT, participants with biochemical hypoglycemia had higher levels of insulin sensitivity, as evaluated by Matsuda ISI. These participants also had higher levels of insulin secretion, both in the early and late stages of insulin secretion, which were evaluated by Stumvoll early and late secretion indexes.

This study was the first epidemiology study in such a large Chinese population that focused on postprandial hypoglycemia. As mentioned above, there were only a few previous studies focusing on reactive postprandial hypoglycemia, and few patients were included in each study. The recent research in a United Kingdom multiethnic population included more than 6000 participants, which is a significant number of patients; however, in that study, the researchers could only calculate HOMA-IR and QUICKI as the indexes for insulin resistance because only fasting and postprandial 2-hour glucose levels and insulin levels were recorded. In our study, with the recorded levels of both glucose and insulin at the time of fasting, also OGTT 30 minutes and 2 hours, a number of indexes were calculated to evaluate the level of insulin resistance as Matsuda ISI, and insulin secretion as the early and late stages of insulin secretion, Stumvoll early and late secretion indexes.

Of course, as a study, there were some limitations. First, we calculated different surrogate indexes of insulin sensitivity as well as insulin secretion, but it was well known that the gold standard should be represented by clamp-derived indexes. Unfortunately, in this large sample epidemiology study, the clamp test could not be made and these indexes could not be collected. Second, in terms of insulin secretion and sensitivity, there are very strong ethnic differences; however, just because this study was based on the data from the cross-sectional study in China, not in other ethnicities, we may not answer this ethnicity difference well.

With the whole evaluation, we confirmed that in a Chinese population, biochemical postprandial hypoglycemia was associated with higher levels of both insulin sensitivity and insulin secretion.

## Figures and Tables

**Table 1:** Phenotypes of biochemical hypoglycemia compared with NGT.

	PPG ≤ 3.0 mmol/l	PPG > 3.0 mmol/l	*p*
*n*	141	26,465	—
Sex (male percent)	55%	39%	≤0.001
Age (years)	42.5 ± 13.1	40.2 ± 12.7	0.03
SBP (mmHg)	119.3 ± 17.9	118.7 ± 15.4	0.66
DBP (mmHg)	77.2 ± 11.1	77.4 ± 12.3	0.84
BMI (kg/m^2^)	22.8 ± 3.2	23.6 ± 3.6	0.01
Weight (kg)	62.0 ± 11.5	62.0 ± 11.3	0.96
Waist (cm)	79.5 ± 9.8	80.3 ± 10.5	0.36
WHR	0.84 ± 0.07	0.84 ± 0.09	0.79
FPG (mmol/l)	4.62 ± 0.65	4.95 ± 0.55	≤0.001
Glu30 (mmol/l)	7.82 ± 2.07	8.09 ± 1.74	0.06
Glu120 (mmol/l)	2.92 ± 0.07	5.69 ± 1.10	≤0.001
SUA (*μ*mol/l)	200.5 ± 148.	260.0 ± 117.7	≤0.001
CHO (mmol/l)	4.60 ± 0.96	4.69 ± 0.91	0.25
TG (mmol/l)	1.41 ± 0.98	1.36 ± 0.94	0.55
HDL-C (mmol/l)	1.34 ± 0.34	1.36 ± 0.36	0.52
LDL-C (mmol/l)	2.68 ± 0.83	2.67 ± 0.77	0.90

**Table 2:** Characteristics of insulin secretion and insulin resistance of biochemical hypoglycemia compared with NGT.

	Glu120 ≤ 3.0 mmol/l^∗^	Glu120 > 3.0 mmol/l	*p*
*n*	141	26,465	—
Fasting insulin (*μ*U/ml)	7.4 (5.0–8.9)	8.1 (5.0–9.5)	0.18
Ins30 (*μ*U/ml)	53.0 (27.6–64.9)	47.9 (22.8–60.1)	0.12
Ins120 (*μ*U/ml)	16.3 (7.5–17.7)	32.4 (14.6–39.7)	≤0.001
Masuda ISI	10.72 (7.44–12.94)	8.64 (5.55–10.79)	≤0.001
Stumvoll early	1338.4 (1162.6–1466.3)	1012.3 (792.1–1180.1)	≤0.001
Stumvoll late	340.8 (310.8–354.8)	260.7 (212.3–294.8)	≤0.001

^∗^The levels were expressed as median (25%–75%); NGT: normal glucose tolerance; Glu120: OGTT 2-hour glucose; Ins30: serum insulin level at 30 minutes after an OGTT; Ins120: serum insulin level at 120 minutes after an OGTT; Masuda ISI: the Matsuda's insulin sensitivity index.

**Table 3:** Association of Masuda ISI, Stumvoll early index, and Stumvoll late index, with biochemical hypoglycemia in screened patients by multivariate logistic regression analysis.

	Odds ratio	95% CI	*p*
Fasting insulin	0.959	0.915–1.006	0.09
Ins30	1.002	0.998–1.005	0.41
Ins120	0.927	0.911–0.943	≤0.001
Masuda ISI	1.075	1.047–1.104	≤0.001
Stumvoll early index	1.002	1.001–1.002	≤0.001
Stumvoll late index	1.007	1.006–1.008	≤0.001

Adjusted by gender, age, BMI, serum uric acid level; Ins30: serum insulin level at 30 minutes after an OGTT; Ins120: serum insulin level at 120 minutes after an OGTT; Masuda ISI: the Matsuda's insulin sensitivity index.
